# Draft Genome Sequence of *Methylobacterium* sp. Strain ARG-1 Isolated from the White-Rot Fungus *Armillaria gallica*

**DOI:** 10.1128/genomeA.00398-16

**Published:** 2016-06-02

**Authors:** Caitlin Collins, Caitlin Kowalski, Jessica Zebrowski, Yevgeniya Tulchinskaya, Albert K. Tai, Magdalena James-Pederson, Rachel Hirst

**Affiliations:** a Department of Biology, Stonehill College, Easton, Massachusetts, USA; b Department of Integrative Physiology and Pathobiology, Tufts University School of Medicine, Boston, Massachusetts, USA

## Abstract

*Methylobacterium* sp. strain ARG-1 was isolated from a cell culture of hyphal tips of the white-rot fungus *Armillaria gallica*. We describe here the sequencing, assembly, and annotation of its genome, confirming the presence of genes involved in methylotrophy. This is the first genome announcement of a strain of *Methylobacterium* associated with *A. gallica*.

## GENOME ANNOUNCEMENT

*Methylobacterium* species have been isolated from a variety of substrates, including, but not limited to, soil (1), air (2), freshwater (3, 4), lake sediments (5), and plants (6). They have been found in association with >70 different types of plants (7), and the association can be symbiotic (8), epiphytic (9), or endophytic (10). Members of the genus *Methylobacterium* are capable of growing on C_1_ compounds, such as methanol (11), and the utilization of methanol released from plant stomata by these organisms has been well documented (12). Although methylobacteria are ubiquitous in nature and predominant in the phyllosphere (13), to date, there have been no reports of *Methylobacterium* species isolated from white-rot fungi, which degrade lignin. Here, we describe the whole-genome shotgun sequence for *Methylobacterium* sp. strain ARG-1, isolated from a hyphal-tip cell line of the white-rot fungus *Armillaria gallica*. A comparison of its 16S rRNA gene using EzTaxon (14) showed high identity with *Methylobacterium brachiatum* B0021^T^ (99%).

Genome sequencing using the MiSeq platform with 250-bp-end reads generated 6,304,876 bp, with an average G+C content of 69.1% and 259× coverage. Sequence trimming (26 nucleotides from the ends) and *de novo* assembly were performed using Edena (version 3.130110) (15, 16), generating 92 contigs (largest contig, 458,130 bp; *N*_50_, 135,077 bp). The draft genome was annotated using both the Rapid Annotations using Subsystems Technology (RAST) server (17) and the NCBI Prokaryotic Genome Annotation Pipeline (PGAP) (http://www.ncbi.nlm.nih.gov/genome/annotation_prok/) (18). A total of 6,003 genes were identified through PGAP and categorized into 5,700 coding sequences, 236 pseudogenes, 4 complete rRNAs (5S and 16S), 51 tRNAs, 1 noncoding RNA (ncRNA), and 89 frameshifted genes.

The genes essential for methanol oxidation were identified on contigs 40 (*mxaCKLEHB*)*,* 66 (*mxaFJGIRSA*), 2 (*mxcQE*), and 4 (*mxbDM* and *pqqABC* or *pqqDE*) (19). There are two duplications of *mxaF*, which is essential for C_1_ growth, resulting in three intact *mxaF* genes found on contigs 1, 4, and 66. In addition, *pqqA*, which is not essential for C_1_ growth, has been duplicated and was found on contigs 4 and 7. Interestingly, the gene encoding the enzyme gamma-4-carboxymuconolactone decarboxylase catalyzing the conversion of 2-carboxy-2,5-dihydro-5-oxofuran-2-acetate to 4,5-dihydro-5-oxofuran-2-acetate as part of the *ortho*-cleavage pathway for bacterial lignin degradation was found in the genome (contig 6) (20). However, other genes encoding enzymes required for this pathway (e.g., protocatechuate 3,4-dioxygenase, beta-carboxymuconate lactonizing enzyme, and beta-ketoadipate enol-lactone enzyme) were not identified. As such, *Methylobacterium* sp. ARG-1 is unlikely to degrade lignin. However, white-rot fungi release methanol during lignin degradation, potentially providing a consistent but competition-reducing substrate for *Methylobacterium* growth (21). Methanol-utilizing bacteria have in fact been isolated from both wood inoculated with white-rot sulfur tuft mushrooms (*Hypholoma fasciculare*) and on the rhizomorphs of *H. fasciculare* while other bacterial growth was suppressed (22). *Methylobacterium* sp. ARG-1 may experience a significant selective growth advantage from its ability to utilize methanol, as it was isolated from hyphal tips of *A. gallica*, a lignin-degrading fungus. The genome data presented will allow for future comprehensive comparative genomic analysis that will begin to elucidate the mechanism for the fungal-bacterial association.

### Nucleotide sequence accession numbers.

This whole-genome shotgun project has been deposited at DDBJ/EMBL/GenBank under the accession no. LHCD00000000. The version described in this paper is version LHCD01000000.
